# Toxic Nightlife Relationships, Substance Abuse, and Mental Health: Is There a Link? A Qualitative Case Study of Two Patients

**DOI:** 10.3389/fpsyt.2020.608219

**Published:** 2021-01-21

**Authors:** Sandra Racionero-Plaza, Juan Antonio Piñero León, Manuel Morales Iglesias, Leire Ugalde

**Affiliations:** ^1^Department of Sociology, University of Barcelona, Barcelona, Spain; ^2^Mental Health Unit, County Hospital of Antequera, Malaga, Spain; ^3^Department of Didactics and School Organization, University of the Basque Country, Bilbao, Spain

**Keywords:** substance use disorder, peer group, intimate partner violence, toxic relationships, mental disorder (disease), psychiatric rehabilitation

## Abstract

**Introduction and Aims:** This article explores the role of toxic close relationships in night life on substance use disorders and mental health conditions. We also contrast the quality and effects of social relationships when doing drugs with those produced by a mental health program that fosters quality relationships between patients.

**Design and Methods:** This qualitative case study was carried out at a mental health day care center of a hospital in Malaga (Spain). The cases of two patients with severe mental disorders and a history of drug addiction were analyzed. Data were collected through in-depth interviews with every patient, semi-structured interviews about each patient with the psychologist of the medical team of the program, and medical documentation. The analysis involved a combination of inductive and deductive approaches.

**Results:** The analysis of the data revealed, on the one hand, the influence of toxic relationships in nightlife, including violent sporadic sexual relationships, in the initiation and persistence of substance use that took part of the mental health disorder in these patients. On the other hand, the findings show that these participants' current involvement in a mental health program, which fosters quality relationships between patients, has brought emotional benefits to both of them.

**Discussion and Conclusion:** This paper points out the relevance of considering quality of social relationships when examining substance use disorders and related mental health problems. Additionally, the findings indicate the importance of fostering quality peer relationships in mental health rehabilitation programs addressed to patients with histories of drug addiction to improve treatment outcome.

## Introduction

### Quality of Social Relationships and Health

Science has well established the impact of close social relationships for individuals' health and well-being (1). Relationship quality can affect immune function, regulation of stress, mood, motivation, coping skills, eating and exercise habits (2), or endocrine function and nervous system activity (3). Scientific literature shows how, depending on their quality, toxic or edifying relationships can affect health in a negative or a positive way.

A close social relationship that includes any form of violence is toxic and deteriorates at multiple levels the mental and physical health of the person who suffers (4–7). On the contrary, it has been evidenced that quality intimate relationships can increase life satisfaction (8), reduce gray matter density within the right dorsal striatum (9), lead to healthier biological profiles (10), lower ambulatory blood pressure (11), better immune functioning (12) and can reduce the harmful effects of stress on the immune system (13), raise higher levels of oxytocin in circulating plasma (14), and they may attenuate responses to chronic pain (15). A study on the impact of close social relationships on mortality found that those individuals with adequate social relationships had a rate of survival 50% higher than those whose social relationships were poor (16). Thus, close social relations are seen as extremely necessary for individual's health, development, and survival through the entire lifespan (17).

### Peer Group Relations and Substance Abuse in Nightlife

The involvement of social relationships in substance use and abuse has also been documented in research. There are studies that point out that the individuals learn the risk-taking behaviors, such as substance use, within the groups of peers (18) or with sex partners in the case of injecting heroin (19). Substance use is related by some to bonding with friends, gain status among peers, and trying to escape and forget (20). Others see it as a relatively inexpensive pastime with friends and as a possibility for intimacy (21).

Collected evidence also suggests that the relationships developed on the basis of substance use can provide, at times, spaces for mutual exploitation, deceit, and distrust (22). The selection of individuals who use drugs as friends increases the availability of substances, and this can lead to progression to potentially more damaging illicit substances (23). For some substance users, friendship contains expectations of substance sharing and reciprocation, purchase for friends and introducing them to dealers—who that way become “friends,” too (24). Yet the quality of all these social relationships that are contexts for drug initiation and addiction have been little explored in depth and even less in cases of patients with SMD whose condition involves a history of drug abuse in night life settings.

Regarding environments, particular settings also increase the likelihood of troubling and toxic relationships, and drug abuse; that is the case of nightlife spaces. In these environments, individuals have different and contradictory experiences of pleasure and harm, such as enhanced and troubled friendships (25). Nightlife is shown to offer many opportunities for novel psychoactive drugs use (26–28). The relationship between the styles of music played in a particular context and the substance use has also been studied (29), or the role of a specific international nightlife resort (30), or music festivals (31) in the substance use. Substance use in nightlife experiences has also been found in correlation to violence (32).

### Substance Use Disorders and Mental Health

Scientific evidence shows that many individuals who develop substance use disorders are also diagnosed with mental disorders, such as depression, anxiety, and post-traumatic stress disorder (33). Likewise, studies of comorbidities have indicated that substance abuse co-exists with mental health disorders, such as anxiety disorders, depression, bipolar disorder, attention-deficit hyperactivity disorder, psychotic illness, borderline personality disorder, antisocial personality disorder, or schizophrenia (34). Recent studies have also advanced this line of research by indicating that there are patients with serious mental conditions emerging from substance abuse (35). These studies have provided a diagnostic framework that offers reliable, unambiguous clinical criteria to differentiate between comorbid conditions, i.e., cases of patients with serious mental disorders who also have a substance use disorder, and substance-related psychoses (35). Achieving this differentiation in diagnosis has important clinical implications.

The picture that all the reviewed evidence provides is of a connection between substance addiction, toxic relationships in night life, and mental health problems. In a time when social relations have come to the front in psychological and medical research, even seeing quality close relations as “life saving” (1), we must inquiry the role played by social relationships of different quality, toxic or quality, in drug initiation and addiction and their related mental health harms. In addition, taking into account the power of social relationships in “saving lives,” we should examine too how quality relationships may aid reversing the psychological damage associated to drug relational experiences. Our study contributes to that scientific endeavor. We examine the characteristics of close social relationships that take place around drug consumption and addiction in young adults who developed SMD as a result, among others, of those experiences, and we do so qualitatively, from the patients' perspective, attending to their views, beliefs, and cognition on this topic, and always in relation to their own experiences. This qualitative inquiry provides “rich description” that has the implication of informing mental health programs or harm reduction interventions in substance use disorder with evidence on the importance of working the quality of close social relationships for those interventions gaining effectiveness.

### Context of the Study

The study was conducted at the Mental Health Day Care Centre of a public county Hospital in Malaga (Spain). The psychiatrist that is the head of the service decided from the very beginning of the Unit, which was by the end of 2008, to overcome a medicalization approach in the mental care provided and to design all attention to the patients based on fostering the quality of peer relationships, and more specifically, the social support of friends, as a main therapeutic tool to treat severe mental illness. While this psychotherapeutic approach was in place since 2008, it was in 2010 when it acquired a more formal status. This orientation is in line with research in the field of psychiatry, which has evidenced the benefits of therapies addressed to increase the quality of peer relationships and to strengthen the structure of social networks to prevent and mitigate symptoms of depression and, more broadly, emotional distress (36, 37). Along these lines, the psychotherapeutic approach implemented in the Day Care Centre includes activities in the hospital, as well as in the community. Examples of these activities are workshops in groups where the patients share feelings, plans, and opinions related to diverse social and personal issues always in a climate of confidentiality, support, and respect, as well as trips to the community and to other towns to visit museums, libraries, theaters, factories, etc, to perform group learning activities. This relational and community-based psychotherapeutic approach also involves developing group meetings in the houses of some patients.

The therapeutic program also implies an egalitarian relationship between patients and the professionals that compose the team of the Unit, thus enhancing supportive relationships between them, overcoming traditional approaches in which hierarchy is more present in the doctor–patient relationship. The program is implemented by a team of professionals composed of a psychiatrist, a psychologist, an occupational therapist, an occupational supervisor, and a nurse. All of them work in a group to design the activities, monitor the patients, and make adjustments in the program.

## Materials and Methods

### Participants

This article reports the findings from the study of the cases of two patients involved in the aforementioned program. The selection of these patients responded to the criteria of: (a) being involved in the therapeutic program with a community and relational approach in the Mental Health Unit, County Hospital of Antequera (Malaga, Spain); (b) having a diagnosis of severe mental disorder, and (c) a history of drug abuse that was involved in the emergence of their mental health condition as reported by the medical team attending to the patients and in their clinical histories. Among all patients involved in the mental health program, it was RTV and JM who were the ones who met the criteria. The patient information that follows was collected through interviews with the psychologist in the program who attends to RTV and JM, and via documentation.

RTV was 32 years old at the time of the interviews and had been participating in the mental health program for 6 years. He has a diagnosis of paranoid schizophrenia and schizoid personality disorder. RTV started taking drugs at 16–17 years old, and the biggest peak of consumption was between 19 and 22 years, when the mental health crisis appeared. RTV is from a small village where everyone knows each other. It was when he went to high school that he entered the drug world. The psychologist explained that RTV was very introvert as an adolescent and blamed himself for being so; he had very poor self-stem and saw himself less valuable than his peers. For all these reasons, RTV felt “out,” excluded from the group. So, according to the psychologist, “when he (RTV) used drugs, he went from being isolated, suspicious, distant, closed, to open up to life. Then, he went out to parties, went to the nightclubs, listened to electronic music and consumed acids.” RTV experienced a crash associated to substance abuse, and the mental health disorder appeared. Profound difficulties with communicating ideas and emotions, and with feeling confident were all manifested in the interviews with RTV. Nonetheless, the medical team states that among all patients with the most serious mental disorders, RTV is the one whose advance has been greatest as a result of participating in the mental health program. The psychologist explained that RTV spent years in the center with almost communicating no word, and now he is able to even express emotions and feelings.

JM was 35 years old at the time of the interview and had participated in the mental health program for 3.5 years. His diagnosis is schizoaffective disorder. From the interview with the psychologist, we knew that JM's mental disorder relates to an important family conflict related to his homosexuality and other traumatic child experiences. JM's father did not accept JM's homosexuality, and JM suffered unceasing psychological abuse from him. With time, JM felt psychologically overloaded, emotionally overwhelmed with such maltreatment, which added to the experience of physical abuse during childhood. When JM entered the mental health day care center, he showed a major risk of suicide and tried to commit it with pills. JM's addiction to drugs during youth was related to feeling attracted to a man who mistreated him psychologically, with whom he had a sporadic relationship. This man played with JM's feelings using his homosexuality in so doing. The psychologist explained that given the abuse in the family because of JM's sexual orientation, he did not want to come out in the open as he was afraid of suffering even more. Going out with people JM came to know at his job, some of whom were gay too, and doing drugs with them, “facilitated” the coming out process for him, and when going to parties with that group and with the man he was attracted to, JM believed he was the person he wanted to be and was not in the family context. The use of drugs made JM feel and behave on the basis of an idealization of himself, to approach that ideal of who he wanted to be. Involvement in the mental health program has provided many benefits for JM, who accepts himself much more now as a result of the sincere recognition from the rest of the patients. This has improved JM's emotional wellbeing.

RTV and JM are both highly engaged in the mental health program with a community and relational therapeutic approach implemented in the Unit. RTV and JM know each other from the group and fully participate in the trips, group meetings, and other community-based activities developed in the therapeutic program. Both RTV and JM enjoy the activities and have good relationships with the rest of the patients who are also engaged, as well as with all the professionals in the Mental Health Unit. As reported above, RTV and JM experienced toxic relationships in early youth, both of them in the peer group with which they went out in the night and one of them also in a sporadic sexual-affective relationship with one man in those groups. Both RTV and JM initiated substance use in the context of those toxic relationships, shared the function that such consumption had for them (to deal with the harassment experienced and to “forget” it), and both had the first psychotic episode in such context. Also, the involvement in the mental health program with a community and relational approach has benefited both RTV and JM; their emotional wellbeing has improved in a community of peers based on trust and respect.

### Data Collection

Data was collected through life story interviews and in-depth semi-structured interviews with the two patients, as well as in-depth, semi-structured interviews about each patient with the psychologist of the medical team of the program who visits RTV and JM. Data collection took place in a room in the mental health day care center of the hospital, a familiar setting for the patients. Collection endured until a point of saturation has been reached. The psychologist was present in the interviews with the patients after asking the patients if they preferred him to be present or not. According to the communicative methodology (38, 39) employed in the research, this possibility was given in order to achieve highest comfort in participants during the interview, something especially relevant in this case given their mental health condition. The psychologist only intervened to rephrase some questions for the patients when it seemed necessary to adjust them to facilitate understanding. The interview protocols addressed to patients included three sets of questions: contextual questions, youth experiences with drugs and their link with social relations, and current social relations in the program and their emotional benefits in comparison to past toxic relationships. The interview protocol used with the psychologist included two very open questions about the diagnosis of every patient and their life stories in relation to their mental disorder and experiences of substance abuse.

### Analysis

The analysis was informed by theories about (a) high-quality relationships and mental and physical health, and (b) preventive socialization of gender violence. Yet the approach was opened enough to incorporate emerging categories for the particular case of drug addiction and nightlife. The interviews were transcribed and analyzed thematically. The purpose of the life stories was to make patients reflect upon their experiences from the past and present on issues such as close social relationships and their influence in substance abuse, nightlife experiences, mental health issues, and the current benefits of the mental health program with a relational approach regarding reversing the negative impact of those social experiences related to drugs. The interviews with the psychologist from the medical team contributed data regarding diagnosis and medical information; he shared the life trajectory of the patients from the perspective of traumatic experiences, relationships linked to the trauma, and mental health issues that evolved with those. The psychologist also shared his perspective on the impact of the mental health program on every of the patients' emotional well-being. Initially, there was a list of 10 descriptive codes informed by prior research and scientific studies on the topic of quality relationships, night life, addiction and the peer group, and the benefits of relational therapy. Five more descriptive codes were included to capture other topics discussed by participants.

### Ethics

This study was fully approved by the Ethics Committee of the Regional Government of Andalusia. It complies with all the guidelines and principles of the Declaration of Helsinki. Participants signed an informed consent after an oral explanation of the study and receiving written information, and had the opportunity to ask questions.

## Results

### Meaning for Doing Drugs: Feeling Disinhibition to Be “In”

Doing drugs was related in both patients with nightlife and, more particularly, with the social interactions in those contexts. RTV felt attracted by the night environment related to concerts of techno music: “I liked those vibes, that environment,” although he realized he had idealized it: “I had it a little idealized,” as the relationships were not what appeared to be. For JM, it was through the work-colleagues, who were very frequent in clubbing where drugs were always present, that he entered such world. Some of those peers were also homosexuals, and JM felt good with those invitations as this favored him feeling more who he “wanted to be.” The group used drugs to be awake and thus to be able to continue partying with almost no sleep:

It was finishing work, and saying: Come on, let's go party! And let's go to this party, and let's go to the other party. […] You would get home and if you slept, it was for an hour (JM).

JM idealized those colleagues, who he saw as cool, giving them more social status.

Both patients considered drugs as an instrument to feel uninhibited in those contexts, as a way to integrate into those idealized groups. They believed that drugs made them behave in ways they thought would favor their acceptance in the group, being more talkative with other members and with other unknown people in the clubs. In RTV's words, “it was a way to feel integrated, it helped me a bit in the relationship, it uninhibited me.” JM explains, “Well, I felt integrated, because it has always been hard for me to engage with other people. Integrated because I spoke with one, I spoke with the other. That was cool.” Moreover, they describe those moments of doing drugs as having fun because they did not perceive themselves good enough to have fun without it. RTV recalls, “I was having a good time, I had fun. If I did not consume, I did not have fun.”

### Superficial and Instrumental Relationships

The patients, through dialogue with the researcher, described the relationships in those night experiences as “instrumental”: they were just a tool to access drugs. There was neither solidarity in the groups, nor other characteristics which define true friendship. JM explained that “It was all superficial. Because it was, of course, for consumption: since I had, you had. So you interested me, and I was interested in you as friends for the same reason [drugs].”

Both patients also shared the important role played by the leader of the group in deciding membership and, in controlling all relationships in the group. According to the narratives of the patients, the leaders only granted membership in the ground of providing access to drugs. This led JM and RTV to continually and increasingly feel the threat of being excluded from the group, so they behaved submissive to the mandates from the leaders. In the words of JM:

There was a risk to be kicked out of the group if they did not behave as expected from them: “[the leaders of the group] well, they were the ones who had the initiative; this one comes in or does not come with us” (…) But I kept quiet. I knew I could be the next one who did not go with them.

### Violent Sporadic Sexual-Affective Relationships and Drug Addiction

Sporadic sexual-affective relationships in the night life settings attended by JM and RTV were mostly violent and linked very much with substance abuse. Those relationships had a very negative effect, particularly on JM's mental health. JM was in love with one of the group leaders who told JM that he was gay too and behaved with JM in ways that made JM believe that person was sexually interested in him in constructive ways. However, that leader never engaged with JM sexually, but mistreated JM every time more. The toxic leader flirted and had sex with girls and boys from the group in front of JM and despised JM in verbal and non-verbal acts. JM felt excluded and sexually rejected, and he consumed more drugs telling himself not to care about such mistreatment. The worse the toxic leader treated JM, the more JM's loss of control in consuming drugs:

He hooked up with other friends of the gang. […] And I saw it. So, then, I consumed more and said: I do not care. (…) I continued to consume more, because at least, in those moments, when he did that, it was what I told myself: look, get over it because all will be forgotten.

Yet the drugs did not help JM to forget the toxic person: “No way. It made the problem even bigger.”

JM manifested consciousness in the interview about the direct link between his attraction toward the toxic leader and his drug addiction; he consumed because he thought that it would help him intimate with that person, but it did not help. Instead, he would suffer publicly the mistreatment of the other person:

Researcher: Was the use of drugs related to this relationship?

JM: Well, yes. Because I wanted to be with him, I accepted all and everything he wanted. (…) In front of friends he did not want to interact with me, as I am gay, but he did not want that to be public.

JM thought that the toxic leader scorned him publicly because JM was gay, and such mistreatment was a way for the leader of hiding that he was gay too.

The psychologist corroborated that JM was in a situation of low self-esteem, in which the bad relationship with his father—that did not accept his sexual orientation and would constantly abuse JM psychologically for that reason–, nightlife and substance use contexts, all these issues made him enter this toxic relationship and accept all the humiliation and mistreatment from the man he liked from the group. In his own words:

When you are in a relationship, in which a person draws your attention a lot and you want to please that person above your health, like this man, you do whatever it takes.

### Benefits of a Mental Health Program Focused on Quality Human Relationships

Since they joined the mental health program with a relational approach at the hospital, RTV and JM discovered what friendship really is, and they have experienced the psychological benefits of quality human relationships. They have gone through unconditional acceptance from their peers and the professionals in the program, with no need to become someone else. This has led RTV and JM to increased emotional well-being.

RTV describes this feeling of unconditional acceptance as making him feel relief: “Relief, that they accept me as I am. And thus, I like to go out and socialize.” JM talks about feeling true affection, non-instrumental, from the other participants, and this feeling makes him feel safe, confident, loved:

Here when you cherish, you cherish. […] we look one after the other. […] Both professionals and colleagues, when they do something for you, is because they love you. (…). I feel safe. I feel confident, like at home. I feel loved, listened.

The quality of human relationships being constructed in the program, based on confidence, make the patients feel less anxiety, be calmer. Such emotional state favors JM and RTV to focus more on people who treat them well and invest in cultivating friendship with their peers. All so raises their feelings of being loved and personal growth. In the words of JM:

I am calmer, […] more centred toward the peers who are looking one after the other, I feel more the affection, a lot of affection. I am loved at home, but here I receive another affection, more special. And very special friends. (…) Helping each other has helped me grow as a person.

The psychologist talked about RTV and JM's poor self-esteem and acute necessity of feeling integrated and being accepted in the group, and how much that played a role in their past toxic experiences. Such need is now healthily met in the mental health program. The psychologist talked about the importance at present, for patients with such condition to get to know the rest of the patients in the group and, from there, express the whole range of human emotions and decrease the pressure of being perfect in order to be accepted by others. The professional reflected on RTV in this regard:

It helps a lot that the others [patients] are able to comment their issues in the open, talk about their difficulties. I think that knowing “the other” gives him [RTV] confidence. For a person, from my point of view, so withdrawn, so distrustful, so injured by life, he has to know you first. He has to listen to you saying a lot, and something loving. (…) I think that has helped him. (…) Because problems are welcome, difficulties are welcome. We listen to them, we talk to them, we learn. It helps you to get rid of that idea that in order to be valid you have to be perfect, that you cannot have fissures.

When talking about JM's case, the psychologist pointed out JM feels for the first time the possibility of being accepted by the group just the way he is, with no need to be under the effects of drugs to be “in.” That made JM gain self-confidence and this, in turn, being more prone to talk with others about his mental and life issues:

For him (JM), it means to be in a place where he does not have to do anything to be accepted, but he is good the way he is. He does not have to simulate anything, he does not have to consume, he does not have to be the most… because he is good the way he is, and we value him for what he contributes [to the group], for what he is. And if there is something that we disagree, well, that's fine. The group welcomes him very well from the beginning. And he is gaining confidence that allows him to speak after a long time.

## Discussion

Research has shown that toxic close relationships can influence physical and mental health for worse (4–7), and quality close relationships can be “life-saving” (8, 40). With qualitative evidence and a focus on drug abuse, this article contributes to the field of research on close relationships and health, mental health more particularly.

Through the cases of two patients with a drug addiction record and mental health disorder diagnoses, we have seen how certain toxic relationships in nightlife might foster substance abuse, which could relate, in some cases, with the development of severe mental disorders. On a positive note, we have seen that a mental health program with a relational approach that promotes high-quality relationships among patients has promoted the emotional well-being of people with serious mental disorders and a history of addiction and toxic relationships in night life. Concretely, mental health programs focused on developing and cultivating quality relationships among patients can be a context in which to address the impact of past toxic relationships associated to drug abuse, providing gains in confidence, self-steam, and social contact, which are central psychological benefits for patients with psychosis and schizophrenia.

Literature in the area of drug and alcohol abuse has established that friends or sexual partners played an important role in both initial introduction to opiates and in the switch to injecting (18, 19). Our study provides qualitative data showing that the quality of those relationships with peers and sexual partners is central to understand introduction to substance abuse. It is in toxic close relationships in party settings where that occurred for the case of the patients analyzed. The study conducted shows particular traits of the relational context that favored drug consumption, and which serves to qualify such relations as toxic and poor, for example, the instrumental use of members in the peer group: they were accepted or rejected, valued or dismissed, depending on whether providing drugs or access to drugs. This is consistent with other research in which consumers of marijuana defined friendship in relation to expectations on sharing and reciprocation (24, 41). In our case, we have shed light too upon the emotional harm that such instrumentality had in the two patients.

Our results also reiterate qualitatively what other studies in the field of substance addiction had found: that youth can use drugs to bond with “friends,” to gain status among peers, and to escape and forget (20–23). Apart from this, our research sheds new light on some personal and sociocultural meanings behind such motivations: to be accepted by group leaders who control membership in the group, and to escape and forget about the mistreatment suffered from violent sporadic sexual partners and peers.

Regarding this last aspect, the findings on the sexual-affective area add more evidence on the much violence present in sporadic sexual affective relationships in night life settings, something which is being explored by the literature on gender violence victimization (42). Yet, our data advances this knowledge, and knowledge on close relationships and health more generally, by crucially showing that such violent sporadic sexual-affective relationships can impel drug consumption and deteriorate mental health in the victims. That is, our results shed new light on more mechanisms operating in such sporadic relationships that explain their violent character, and the impact that they can have on drug use and emotional wellbeing. This finding is central from the point of view of prevention of dating violence among youth.

## Conclusion

Quality of close relationships stands one time more as essential to healthier lives. Our results reiterate with qualitative data from two patients what other quantitative research in the field of addiction had shown: toxic networks of peer friendship groups in which drug consumption take place are likely to be sites of mutual exploitation, deceit, and distrust (22). We do so, although with data from patients with SMD, which allows to shed some light on the potential connection between toxic relationships in the context of drug consumption and worsened mental health outcomes, proving qualitatively that toxic social relations, either peer or sexual-affective, are adverse for the mental health of the group members, even being triggers of mental health disorders for some. Yet quality relationships experienced later in life could contribute to mitigate some of those negative psychic effects and improve the emotional condition of those subjects. This result has important social impact (43): Mental health programs addressed to patients who have histories of drug addiction can be enriched with activities that support the creation and cultivation of quality relationships. Also, drug prevention campaigns tackling youth should incorporate reflection upon the quality of social relationships in night clubbing, and of violent sporadic sexual-affective relationships more particularly, and their role in substance initiation and addiction, so young people learn that close relationships of poor quality in nightlife spaces might well raise their risk to fall into drug addiction and deteriorate their mental health.

In addition, our findings can also inform drug policy on novel psychoactive drugs. Scientific knowledge on the motivation for using novel psychoactive substances is still scarce, particularly from a qualitative method perspective (44). Our study provides information on two users' reasons for doing drugs in nightlife settings, and they both point out to cope with stressful situations derived from toxic close relationships, including violent sexual-affective relationships. In this regard, drug prevention policies can raise their effectiveness in acknowledging the role played by toxic social relations in nightlife settings, more specifically, the attraction to toxic individuals that consumers may feel, as promoters of consumption of novel psychoactive drugs among youth. If taking this into account, these policies will make an important step forward as it has been common to point to substance abuse as a risk factor for violent close and sexual-affective relationships—and much training for youth has taught this—while our results show the inverse direction, that is, toxic relationships can foster substance abuse as a tool to cope with the distress in the relationship while continuing with it.

Our results also have implications for the media. Prior studies on preventive socialization of gender violence have shown the role of the media (TV series, movies, advertisements, video clips, social media, etc) in socializing youth into a *dominant coercive discourse* that presents individuals with violent attitudes and behaviors as more attractive and desirable (45). Disseminating our results among those in charge of producing contents for the media can be useful to raise their awareness about the mental health harm that such dominant coercive discourse can have on the youth, as a first step toward a profound revision of the role of the media on promoting or ending with toxic relationships.

This research has limitations. First, it follows a design of qualitative case study, so these results, despite informative and providing rich description, cannot be generalized. Other studies are needed, which examine relations between violent sporadic relationships, drug consumption, and mental health in large samples of adults who have developed serious mental disorders and had stories of exploitation in relationships in night life and drug consumption. This would allow to deepen the correlations and the link between toxic relationships in night life, substance use disorder, and mental health. Second, longitudinal research would be also of interest to track whether and how patients with severe mental disorders and stories of violent close relationships in nightlife where they initiated the consumption of drugs, and who are now engaged in psychiatric rehabilitation programs focused on cultivating quality relationships improve their emotional wellbeing throughout time for the specific psychological traits damaged in their toxic experiences.

## Data Availability Statement

The raw data supporting the conclusions of this article will be made available by the authors, without undue reservation.

## Ethics Statement

This study, involving human participants, was reviewed and approved by the Ethics Committee of the Regional Government of Andalusia. The patients/participants provided their written informed consent to participate in this study. Written informed consent was obtained from the individual(s) for the publication of any potentially identifiable images or data included in this article.

## Author Contributions

SR-P and JP conceptualized the research. S-RP, JP, and MM did the investigation. SR-P and LU took charge of the methodology. SR-P wrote the original draft. SR-P, MM, and LU reviewed, edited, and wrote the manuscript. All authors have read and agreed to the published version of the manuscript.

## Conflict of Interest

The authors declare that the research was conducted in the absence of any commercial or financial relationships that could be construed as a potential conflict of interest.
